# Transient spleen enlargement in peripheral blood progenitor cell donors given G-CSF

**DOI:** 10.1186/1479-5876-2-25

**Published:** 2004-07-21

**Authors:** David F Stroncek, Kristin Dittmar, Thomas Shawker, Angela Heatherman, Susan F Leitman

**Affiliations:** 1 Department of Transfusion Medicine, Warren G. Magnuson Clinical Center, National Institutes of Health, Bethesda, Maryland, USA; 2 Department of Diagnostic Radiology, Warren G. Magnuson Clinical Center, National Institutes of Health, Bethesda, Maryland, USA

**Keywords:** granulocyte colony-stimulating factor, peripheral blood progenitor cells, splenomegaly, spleen

## Abstract

The administration of granulocyte colony-stimulating factor (G-CSF) to peripheral blood progenitor cell (PBPC) donors causes spleen length to increase, but the duration of enlargement is not known. Eighteen healthy subjects were given 10 μg/kg of G-CSF for 5 days and a PBSC concentrate was collected by apheresis. Ultrasound scans were used to assess craniocaudal spleen length before and after G-CSF administration. Mean spleen length increased from a baseline length of 10.7 ± 1.3 cm to 12.1 ± 1.2 cm on the apheresis day (p < 0.001). Ten days after apheresis, spleen length fell to 10.5 ± 1.2 cm and did not differ from baseline levels (p = 0.57), but in 3 subjects remained 0.5 cm greater than baseline length. Increases in spleen length in PBPC donors are transient and reversible.

## Background

Peripheral blood progenitor cell (PBPC) concentrates donors are routinely given granulocyte colony-stimulating factor (G-CSF) to increase the concentration of circulating PBPCs and hence the number of progenitors that can be collected by apheresis. Typically 10 to 16 μg/kg of G-CSF are given subcutaneously daily for 4 to 6 days prior to the collection [1-3]. The administration of G-CSF to healthy PBPC concentrates donors is very safe, but there have been four reports of spontaneous rupture of the spleen and splenectomy in healthy allogeneic PBPC donors given G-CSF [4-7].

While spontaneous rupture of the spleen in PBSC donors given G-CSF is rare, the administration of G-CSF for five days causes spleen length to increase in almost all healthy donors [8,9]. The increase in length is highly variable, but the mean increase is approximately 13%. Spleen length begins to return to baseline levels quickly, but it is not known how long it takes to return to baseline. In a previous study of 20 PBPC donors given 10 μg/kg of G-CSF for 5 days, we found that spleen length measured four days after the last dose of G-CSF was less than the length on the day of apheresis but greater than baseline values [8].

Since allogeneic PBPC donors may be at risk for splenic rupture while the spleen is enlarged, it is important to determine when spleen size returns to baseline levels. The purpose of this study was to determine if spleen length returns to baseline 10 days after G-CSF-mobilized PBPC concentrates are collected by apheresis from healthy subjects.

## Methods

### Study design

All of the subjects were in good health and were donating G-CSF-mobilized PBPC concentrates for laboratory investigations. The donors were given 10 μg/kg of G-CSF (Filgrastim, Amgen, Thousand Oaks, CA) daily for 5 days, and a PBSC concentrate was collected approximately 2 hours after the last G-CSF dose was given. PBPC concentrates were collected with a CS3000 blood cell separator (Baxter Health Care Corporation, Round Lake, IL). Spleen length was evaluated by ultrasound examination three times: prior to the administration of the first dose of G-CSF, on the day of apheresis, and 10 or 11 days after apheresis. This study was approved by the Institutional Review Board of the National Heart, Lung, and Blood Institute, National Institutes of Health, Bethesda, Maryland.

### Spleen length assessment

Craniocaudal spleen length was assessed using ultrasound (Acuson Aspen Advanced, Siemens Medical Solutions Ultrasound Division, Mountain View, CA) with a sector transducer (Acuson, 4V1, 4.0 mHz frequency, Siemens Medical Solutions). The intra-observer error for measuring spleen length using ultrasound is 4.9 mm when healthy subjects are evaluated at separate settings [10].

### Blood counts and chemistries

Complete blood counts were performed with an automated cell counter (Cell Dyne 4000, Abbott Diagnostics, Santa Clara, CA). CD34+ cell counts were performed using a flow cytometer (Beckman Coulter, Miami, FL).

### Statistical analysis

Spleen lengths measured before and after the G-CSF course were compared using 2-tailed paired t-tests. Spleen length changes were also compared among males and females and Caucasians and non-Caucasians using 2-tailed t-tests. The percent change in spleen length was compared with blood counts, CD34+ cell counts, and donor age using linear regression.

## Results and Discussion

**Figure 1:**
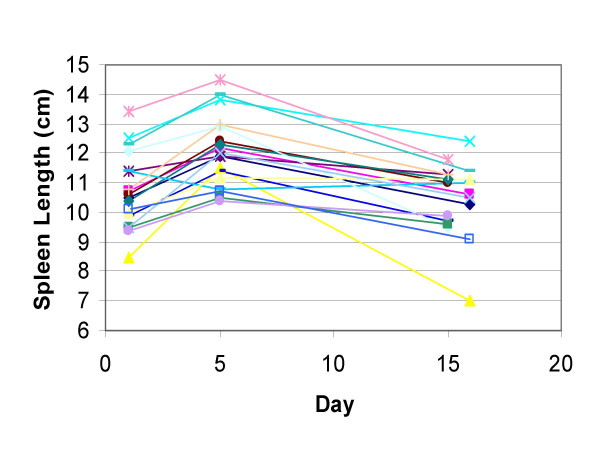
Spleen length changes in healthy subjects donating G-CSF-mobilized PBPC concentrates. Eighteen subjects were given 10 μg/kg of G-CSF for 5 days and a PBPC concentrate was collected approximately 2 hours after the last dose of G-CSF. Spleen length was measured by ultrasound before G-CSF was given (day 1), immediately after the PBPC concentrate collection (day 5), and approximately 10 or 11 days after the collection (day 15 or 16).

The median age of the 18 healthy subjects was 34 years old and ranged from 22 to 55 years of age. Eight of the subjects were male, 13 of the donors were Caucasian, 3 were African American, and 2 Asian. Apheresis day spleen length increased above baseline length in 17 of 18 donors (Figure 1). The mean spleen length increased from a baseline level of 10.7 ± 1.3 cm to 12.1 ± 1.2 cm on the day of apheresis (p < 0.001). The mean increase in length was 1.4 ± 0.8 cm or 13.1 ± 8.9%.

Spleen length was measured 10 or 11 days after apheresis in all 18 subjects. The mean spleen length 10 days after apheresis fell to 10.5 ± 1.2 cm and was less than the apheresis day spleen length (p < 0.001). There was no difference between the 10-day post-apheresis and pre-G-CSF spleen length (p = 0.57). The spleen length 10 days after apheresis was less than the apheresis day length in all 17 donors whose spleen length increased. However, the spleen length 10 days after apheresis remained more than 0.5 cm greater than baseline spleen length in 3 subjects (Table 1). All 3 were Caucasian and 2 were female. Their spleen length remained 6.7%, 11.0%, and 10.5% greater than baseline levels. Two of these subjects had relatively large increases in spleen length of 18.3% and 26.3%, but their spleen length fell considerably 10 days after apheresis. It is likely that the spleen returned to baseline length in these 2 subjects shortly after the third ultrasound was preformed. The other subject's spleen increased only 12.0% in length and 10 days after apheresis had changed little. It is not certain when this subject's spleen returned to baseline length.

**Table 1:** Peripheral Blood Stem Cell Donors Whose Spleen Length 10 Days After Apheresis Remained More than 0.5 cm Greater than Baseline Length

				Spleen Length (cm)			
				
**Donor**	**Age (Yrs)**	**Gender**	**Race**	**Baseline**	**Apheresis Day**	**10 Days Post-Apheresis**	**Enlargement 10 Days Post-Apheresis**
7	23	Female	Cauc	10.4	12.3	11.1	0.7
13	22	Female	Cauc	10.0	11.2	11.1	1.1
14	54	Male	Cauc	9.5	12.0	10.5	1.0

Cauc = Caucasian

PBPC donors with the largest increase in spleen size may be at the greatest risk for spontaneous splenic rupture. In order to determine if subject age, gender, race, or post-G-CSF blood counts affected the magnitude of spleen enlargement, we assessed the relationship between these factors and percent change in spleen length in the 18 subjects in this study and 20 subjects studied previously [14]. There was no difference in the spleen length increase between males and females (12.3 ± 9.7% versus 14.8 ± 8.0%, p = 0.40) or between Caucasians and non-Caucasians (13.5 ± 8.4% versus 13.6 ± 10.4%; p = 0.98). Spleen length increase was not related to donor age (r = 0.13). In addition, spleen length increase was not related to preapheresis CD34+ (r = 0.04), WBC (r = 0.05), neutrophil (r = 0.07), lymphocyte (r = -0.14), monocyte (r = -0.04), and platelet counts (r = 0.19) or hemoglobin level (r = -0.04).

## Conclusions

Healthy PBPC concentrate donors given G-CSF should be warned that their spleens will be enlarged for a brief time and that they may be at risk of splenic rupture. Most donors are likely at risk for splenic rupture only during the time of G-CSF administration and for about 10 days after the completion of the G-CSF course. Since splenic enlargement may persist for longer periods in some donor, until more data are available it may be worthwhile to counsel PBPC donors to avoid activities that could lead to abdominal and splenic trauma for 2 to 3 weeks after the last dose of G-CSF.
